# Successful control of Echinococcosis in Argentina and Chile through a One Health approach, including vaccination of the sheep intermediate host

**DOI:** 10.1017/S0031182024000519

**Published:** 2024-11

**Authors:** Thelma Veronica Poggio, Tomas Chacon, Edmundo Larrieu

**Affiliations:** 1 Instituto de Ciencia y Tecnología “César Milstein”- Fundación Cassara – CONICET, Buenos Aires, Argentina; 2 Servicio Agrícola y Ganadero, Dirección Regional Aysén, Aysen, Chile; 3 Universidad Nacional de La Pampa, Facultad de Ciencias Veterinarias, General Pico, Argentina; 4 Universidad Nacional de Rio Negro, Escuela de Veterinaria, Choele Choel, Argentina

**Keywords:** Cystic echinococcosis, control, South America, vaccine

## Abstract

Cystic echinococcosis control in South American countries requires a comprehensive integrative ‘One Health’ approach. While insular nations have seen successful in their elimination programmes, South American countries face persistent challenges in hostile environments, with *Echinococcus granulosus s.l.*, posing a significant public health concern. Vaccination of intermediate hosts has demonstrated the efficacy of the EG95 vaccine in reducing transmission rates. For example, since 2009, Rio Negro Province in Argentina has added, with marked success, the EG95 vaccine to the control programme, supplementing dog deworming. The Aysen Region of Chile has also reported encouraging preliminary results in reducing cyst prevalence in vaccinated sheep after 3 years of vaccination. The challenges in aligning control strategies with socio-cultural factors, especially in indigenous communities, underlines the need for context-specific strategies. The Rio Negro programme demonstrated commendable compliance, underlining the importance of community engagement in achieving lasting success. The most promising strategies for effective echinococcosis control involved dog deworming and the routine vaccination of sheep and/or goats, underscoring the importance of sustained implementation until all grazing animals have been replaced. For lasting success, these interventions need to be combined with a robust surveillance system.

## Graphical abstract

**Figure:**
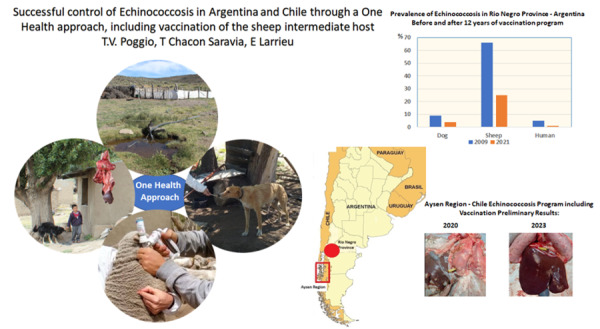

## Introduction

The control of cystic echinococcosis (CE) involves the participation of Animal Health, Public Health, Social Sciences and Research & Technology Organizations and remains one of the best comprehensive strategies of ‘One Health’ approach.

Elimination of echinococcosis have been successful only in insular countries, for example in Tasmania and New Zealand (Craig and Larrieu, 2006; Larrieu and Zanini, 2012). In South American countries such as Peru, Brazil, Chile, Uruguay and Argentina, *Echinococcus granulosus s.l* poses a significant public health concern (Pavletic *et al*., 2017). Several control programmes have been developed in these countries, with varying levels of success and, in some cases, facing challenges for their continuity (Larrieu *et al*., 2019*a*). Therefore, there is an imperative need to validate new models and strategies supporting the ‘One Health’ approach that might be effectively replicated in different South American countries.

The vaccination of intermediate hosts reduces the transmission rate of *E. granulosus s.l*., consequently leading to a reduction in human infections even when control programmes face many practical difficulties (Larrieu *et al*., 2013; Poggio *et al*., 2022*b*).

The recombinant EG95 vaccine has been shown to induce specific antibodies against oncosphere proteins and demonstrated its efficacy in protecting intermediate hosts during trials conducted in New Zealand, Australia, China and Argentina (Lightowlers *et al*., 1999; Heath *et al*., 2003; Heath and Koolaard, 2012; Poggio *et al*., 2016).

In 2011, Providean Hidatec EG95® became the first approved recombinant vaccine for use in sheep, goats, cattle, and South American camelids (Poggio *et al*., 2016; Jensen, 2019). Subsequently, all vaccination programmes in Latin America have incorporated this formulation providing valuable insights into the overall control strategy against the disease in different epidemiological environments.

The EG95 recombinant vaccine trials have used a standardized protocol administering 2 injections, spaced 1 month apart, and annual booster in sheep, goats, and llamas at an early age (Poggio *et al*., 2016). However, some programmes have included adult animals, such as breeding females, in their vaccination strategy (Jensen, 2021).

As the vaccine is equivalent in composition and presentation to the original New Zealand/Australian formula (Lightowlers *et al*., 1996) it has been considered suitable and has been used in control programmes: in Argentina, Chubut Province, 2007–2013 (Poggio *et al*., 2022*b*) and Río Negro Province, from 2009 – to date (Larrieu *et al*., 2013, 2015, 2017, 2019*b*; Labanchi *et al*., 2022); in Chile, Alto Biobio, 2016–2020 (Gädicke *et al*., 2022) and Aysen, from 2020 – to date (Ministerio de Agricultura Chile, 2023) and in Peru 2015–2019 (Larrieu *et al*., 2019*a*; MINSA–SENASA, 2021).

## EG95 vaccine as a new control tool in the South American context

The geographic zone where the programmes are active includes the Patagonian region, shared by Argentina and Chile. The 2 currently ongoing programmes that include vaccination of sheep are located in the Rio Negro Province (Argentina) and in the Aysen Region (Chile).

The Rio Negro Province is home to Mapuche native communities living in communal lands overseen by a religious-political leader known as the ‘Lonco’. The area lacks road infrastructure, animal-handling facilities are basic, and communication technologies are limited to radio as Wi-Fi is unavailable. People often rely on natural water sources for drinking and frequently interact closely with dogs, sometimes sharing sleeping spaces with them. The veterinary and technical teams involved in the control programme have demonstrated impressive non-verbal communication skills and understanding of the culture, customs, and rituals of the local communities, fostering trust. Additionally, in Argentina, a native health worker has served as a crucial intermediary, facilitating communication with residents.

In Chile, the Aysen vaccination area is located in one of the most endemic regions, with very limited knowledge on the transmission of the disease, but the highest rates of hospital admissions and highest incidence rates in children under 15 years of age (Vivanco Concha *et al*., 2021). This is particularly worrying, considering the significant (and common) underreporting of CE cases and the elevated death rates in certain parts of the region (Colombe *et al*., 2017; Medina *et al*., 2021). In this region, the rural population are mostly settlers of mixed origin and land ownership is individual rather than communal.

## Rio Negro province- Argentina vaccination control programme

Since 1980 the Rio Negro control programme for echinococcosis has been based on dog treatment (deworming) with praziquantel (PZQ), carrying out 4 rounds of home visits annually.

Surveillance included serological studies and abdominal ultrasound surveys for children. The programme has been successful in reducing the incidence of echinococcosis in humans and in dogs but not sufficiently to prevent continued transmission of the parasite and the continued incidence of human disease (Larrieu *et al*., 2011, 2019*c*; Larrieu and Zanini, 2012). For this reason, since 2009 Rio Negro province has implemented the vaccine as an additional control measure in endemic rural areas covering approximately 1054 km^2^. The vaccination programme involved 79 farms with vaccinated sheep (10 to 100 sheep per farm), 71 farms as a non-vaccinated control group, and 311 dogs, considering transboundary movement of sheep and dogs. Goats were excluded from the vaccination schedule.

In 2009 and subsequent years, thirty-day old lambs received 2 initial immunizations 1 month apart before weaning and a final booster immunization at approximately 1–1.5 years of age. PZQ treatment to dogs 4 times per year covered both the vaccinated and control areas, and 2 extra deworming rounds were introduced in 2018 (Labanchi *et al*., 2022). It should be noted that the Rio Negro programme faced 2 major challenges: (a) Dogs remain infected despite PZQ treatment every 3 months and (b) dogs roam between the vaccinated and control area (Larrieu *et al*., 2013).

The programme included the EG95 vaccine provided by Melbourne University until 2017, and from 2018 to date, Tecnovax's vaccine is being used. The programme-maintained vaccination coverage was close to 80% for the initial, second, and third vaccination rounds every year from 2009 to 2022 (Labanchi *et al*., 2022).

Following 12 years of using the EG95 vaccine, results showcase the substantial impact of the vaccination programme in reducing the prevalence of the disease, and a sustained increase in EG95 antibody levels in the sheep population (Table 1), (Larrieu *et al*., 2017).

**Table 1.** Assessment of echinococcosis prevalence during 12 year of control programme including dog deworming and vaccination in sheep in Rio Negro Province-Argentina

	2009	2015	2017	2021	Source
**Dog prevalence**					
Arecoline test	4.3%		4.5%		Larrieu *et al*. (2019*b*)
CoproELISA	9.6%		3.7%		
EU with at least 1 infected dog	20.3%		8.9%		
**Rate of infection over 6 y/o animals**					
Necropsy					Larrieu *et al*. (2013)
Non-vaccinated sheep	66.1%^a^				Larrieu *et al*. (2015)
Vaccinated sheep		21.1%^b^			Labanchi *et al*. (2022)
EU with at least 1 infected sheep	84.2%	20.2%			
Non-vaccinated goat				7.1%^c^	
Immunodiagnosis -ELISA					
Non-vaccinated sheep	61.3%^d^				Labanchi *et al*. (2022)
Vaccinated sheep				25%^e^	Poggio *et al*. (2022*a*)
Non-vaccinated goat				30.4%^f^	

Statistically significant results were considered as *P* value <0.05; a *vs* b (*P* = 0.0016); b *vs* c (*P* = 0.23); a *vs* c (*P* = 0.0016); d *vs* e (*P* = 0.0004); e *vs* f *P* = 0.254); d *vs* f (*P* = 0.0013)

Human prevalence also decreased from 5.6% in 2009 to 0.12% in 2015 in the area. Ultrasonography screening showed no symptomatic cases nor any new cases in the period (Larrieu *et al*., 2019*c*). Assessing echinococcosis prevalence in different hosts after 15 years would allow the evaluation of the impact of vaccination.

## Aysen-Chile vaccination control programme

From 1982 to 2001 the Agricultural Livestock Service (SAG) conducted a successful dog deworming programme every 45 days. Unfortunately, funding was not renewed thereafter (Catalán Carvajal, 2007). In 2016, a Livestock Recomposition Transfer Programme started with sheep vaccination in Galera Chico-Balmaceda and El Maitén. After a great drought and the poor general condition of sheep, the programme was discontinued (Jensen, 2021).

The SAG of the Aysén Region has been leading a CE control programme, which includes sheep vaccination, since 2020. It is financed by the Regional Government of Aysén and executed jointly with the Ministry of Health, being a milestone in the fight against CE.

This control programme promotes regional sheep repopulation, by enhancing herd immunity and improving the productivity of farms (Chacon, 2023). It involves the following components:

1. Registration and identification of vaccinated sheep and dogs, linked to epidemiologic units (EU).
2. Administering 1 dose to pregnant sheep and 2 doses for lambs, 1 month apart, in accordance with dental chronometry, clostridial vaccination and deworming.
3. Rigorous slaughter controls.
4. Monitoring EG95 antibody levels.
5. Necropsy on vaccinated sheep.
6. Parasitological diagnostic and quarterly dosing (deworming) implemented directly in the mouth of the dog.
7. A baseline survey about health education among the local population.

The reference population included 23 000 sheep, 1500 dogs, and 1500 goats in 312 EU that correspond to local peasant family farms. The number of EG95 vaccinations administered across the 3 years of the programme demonstrates high vaccination coverage at 89.9% (Table 2).

**Table 2.** Strategies of management in Aysen Region echinococcosis control programme including vaccination and dog dosing. Number of doses received by sheep and dogs and UE concerned (2020–2023).

	Cochrane				Chile Chico			Total
Management practices	2020	2021	2022	2023	2021	2022	2023	2020-2023
Vaccinated breeding female	3856	5970	3937	3831	11.564	8689	3864	41 711
EU with vaccinated adults	38	53	47	49	126	113	49	
Lambs 1st dose	1003	4336	3321	2091	152	2586	2782	16 271
EU with lambs 1st dose	25	130	93	79	15	83	75	
Lambs 2nd dose	25	2366	1894	1689	92	1932	797	8795
EU with lambs 2nd dose	2	79	63	65	13	73	35	
Sheep deworming	8384	15 923	13 486	9407	14 315	11 588	7100	80 203
EU with sheep deworming	77	154	118	83	144	130	49	
Sheep receiving clostridial vaccine	4155	366	1478	468	304	7121	6490	20 382
EU receiving clostridial vaccine	42	8	6	3	10	90	71	
Dog deworming	464	1690	1704	1095	700	1952	1413	9018
EU with dog deworming	111	312	295	190	146	352	250	

It is noteworthy that while the programme aims to include 1500 dogs with 4 deworming treatments per year, achieving this target has proven challenging (Table 2).

The baseline health education survey showed a good knowledge of sheep–dog transmission, but a low level of knowledge of the dog–sheep transmission. Feeding dogs with viscera is common for economic reasons, underlining the importance of further health education and awareness efforts (Sepulveda Valenzuela, 2023). The cultural patterns and structure of peasant family farming in the region highlights the importance of engaging the women who own the house for the periodic deworming of dogs.

After 3 years of programme, encouraging preliminary results after the examination of animal viscera suggests a drastic decrease in the presence of cysts in vaccinated sheep, with the baseline prevalence in old animals as high as 70%. Besides, measurement of vaccine antibody levels has yielded results consistent with the references (Larrieu *et al*., 2017).

Considering a replacement rate of 20%, as older animals are gradually removed by vaccinated ones, and given that the programme is expected to continue for at least 9 or 10 years, it is well placed to achieve comprehensive and sustained success in reducing the prevalence of the disease. (Torgerson, 2006; Poggio *et al*., 2022*b*).

## Discontinued vaccination programmes

The discontinued programmes from Chubut Province – El Chalia Colony (Argentina) and Alto Biobio Region (Chile) left lessons on the receptiveness of rural communities to vaccinating their flocks, and emphasized the importance of assessing the potential negative impacts of stopping these programmes, including the effect on public health goals. Indeed, in the aftermath prevalence can return to high levels when flock immunity is not persistent, and there can extensive social ramifications in different areas (cultural, beliefs, customs, community, rights, security, well-being, fears) that are usually not considered.

In this regard, the underfunded control programme from Alto Biobío Region (2016–2020) included a substantial Pehuenche population engaged in agricultural activities, particularly in breeding sheep and goat flocks. An innovative approach involving veterinarians who provided training to indigenous people-built trust between farmers and the healthcare teams. This training was conducted through a hands-on experiential learning process within the Alto Biobio community (Gädicke *et al*., 2022; Poggio *et al*., 2022*b*).

Therefore, vaccination programmes must consider not only the technical-epidemiological dimension, but also sociocultural understanding and analysis of the context in which they are operating.

## Conclusion

The challenge of implementing straightforward instructions, such as avoiding feeding raw viscera to dogs and ensuring regular dosing, is rooted in complex socio-cultural factors, especially among autochthonous communities (Iriarte, 2019). These cultural patterns are often overlooked in control programmes, highlighting the difficulty in effectively engaging these populations.

Despite these challenges, the Rio Negro programme demonstrated commendable compliance with the sheep vaccination schedule, even under adverse conditions. However, it is essential to recognize that while the vaccine prevents new infections, it does not affect established echinococcal cysts. Therefore, maintaining the vaccination schedule until all grazing animals are replaced is crucial for lasting disease control, emphasizing the need for sustained efforts and community engagement.

‘One Health Programs’ aiming to eliminate CE recognizes that even with the vaccine, it may take 10 years or more to succeed. Once the vaccination programme is over, health education and deworming efforts should be continued. The vaccine, now widely available and produced in Argentina, could be instrumental in potentially eliminating *E. granulosus s.l.* from South America.

The most promising strategies for effective disease control involve dog dosing and routinely vaccinating sheep and goats. However, maintaining the vaccination schedule until all grazing animals are replaced is critical for a comprehensive approach to disease management.

## Data Availability

Not applicable.
